# Modeling the Interictal Epileptic State for Therapeutic Development with Tetanus Toxin

**DOI:** 10.3390/brainsci14070634

**Published:** 2024-06-25

**Authors:** Faezeh Eslami, Arden Djedovic, Jeffrey A. Loeb

**Affiliations:** Department of Neurology and Rehabilitation, University of Illinois Chicago, 912 S Wood Street, 174N NPI M/C 796, Chicago, IL 60612, USA; feslami@uic.edu (F.E.); ardend2@uic.edu (A.D.)

**Keywords:** animal epilepsy models, interictal spiking, therapeutic epileptiform activities

## Abstract

Focal forms of epilepsy can result from a wide range of insults and can vary from focal symptoms to generalized convulsions. Most drugs that have been developed for epilepsy focus on the prevention of seizures. On Electroencephalography (EEG), seizures are characterized by a repetitive buildup of epileptic waveforms that can spread across the brain. Brain regions that produce seizures generate far more frequent ‘interictal’ spikes seen between seizures, and in animal models, these spikes occur prior to the development of seizures. Interictal spiking by itself has been shown to have significant adverse clinical effects on cognition and behavior in both patients and animal models. While the exact relationships between interictal spiking and seizures are not well defined, interictal spikes serve as an important biomarker that, for some forms of epilepsy, can serve as a surrogate biomarker and as a druggable target. While there are many animal models of seizures for drug development, here we review models of interictal spiking, focusing on tetanus toxin, to study the relationship between interictal spiking, seizures, cognition, and behavior. Studies on human cortical regions with frequent interictal spiking have identified potential therapeutic targets; therefore, having a highly consistent model of spiking will be invaluable not only for unraveling the initial stages of the pathological cascade leading to seizure development but also for testing novel therapeutics. This review offers a succinct overview of the use of tetanus toxin animal models for studying and therapeutic development for interictal spiking.

## 1. Introduction

Epilepsy is a common neurological condition characterized by spontaneous seizures and associated with many behavioral comorbidities [1]. Focal epilepsy stands out as a prominent subtype among epilepsies due to its localization within specific brain regions, making it suitable for surgical resections. During seizure episodes, epileptic activity may initially remain confined to this localized area or propagate to involve a larger brain volume, potentially extending across both hemispheres and resulting in a secondary generalized seizure [2,3]. However, seizures are relatively infrequent occurrences, posing challenges in identifying epileptic foci based on seizure onset. Emerging evidence indicates that abnormal brain networks extend beyond seizure events, manifesting more frequently in interictal periods [4,5]. Among these interictal events, interictal spikes are characterized as brief electrographic transients lasting less than 200 milliseconds, featuring a short sharp wave followed by a sustained slow wave [5,6]. In focal epilepsy, interictal spiking does not follow a regular pattern and typically occurs at a frequency of less than 3 Hz. This contrasts with the spike-and-wave complexes seen in genetically generalized epilepsies. Interictal spikes are detected in over 90% of individuals with epilepsy during repeated electroencephalogram (EEG) monitoring [7]. There is evidence suggesting that, following a brain injury, spikes may often emerge before seizures, implying a potential role in facilitating epileptogenesis. This notion gains further support from studies demonstrating that the surgical removal of areas exhibiting high spike activity, along with seizure-generating regions, correlates with improved surgical outcomes [8]. Our understanding of the dynamic network of these events and their relationship to seizures remains limited. While interictal spikes commonly originate from synchronously firing neurons near or at seizure onset zones, they are also observed in cortical regions distant from seizure initiation. Furthermore, beyond their role as biomarkers of epileptic brain regions, interictal spikes may exert an independent impact on behavior. They are found across a spectrum of neuropsychiatric disorders, including anxiety, attention deficit hyperactivity disorder (ADHD), autism spectrum disorder (ASD), obsessive–compulsive disorder, depression, and schizoaffective disorder, even in the absence of seizures [9,10,11,12,13,14].

The hallmark of epilepsy is the sudden, synchronous firing of numerous neurons, leading to various pathological psychomotor manifestations. The mechanisms underlying epileptic activity are complex and involve multiple cellular and molecular processes. One contributing factor is the dysregulation of extracellular potassium levels, which can influence neuronal excitability and seizure propagation. For instance, the astrocytic syncytium helps regulate extracellular potassium, mitigating its impact on neural circuits. Understanding these and other mechanisms is crucial for developing more effective treatments for epilepsy [15]. At present, our clinical inventory for managing epilepsy primarily revolves around medications aimed at suppressing seizures, but they do not offer curative solutions. Despite considerable endeavors, current anti-seizure drugs exhibit restricted effectiveness in halting the development of epilepsy, along with considerable, often life-long side effects [16]. Furthermore, the widespread occurrence of pharmacoresistance and the enduring presence of comorbidities following seizure management emphasize the crucial need for a deeper and more comprehensive understanding of the underlying mechanisms. Although certain anti-seizure drugs have demonstrated efficacy in suppressing spikes, there is currently a lack of medications specifically designed to target spike activity. This enhanced understanding is crucial for driving the development of innovative medications and therapeutic strategies capable of effectively addressing both seizure activity and the associated comorbid conditions [17].

Reliable animal models are critical to facilitate the transition from experimentation to human clinical trials and provide opportunities to enhance our understanding of the underlying pathophysiology and mechanisms of focal forms of epilepsy and interictal spiking. Animal models additionally provide insight into the pharmacokinetics, side effects, potency, efficacy, and tolerance of potential therapeutic agents under investigation. In rodents, chronic epilepsy can be effectively induced through various methods, including the administration of kainic acid (KA), pilocarpine, kindling, and intracerebral injection of tetanus toxin (TeNT). Both the pilocarpine and KA models involve the administration (either systemic or intracerebral) of chemoconvulsant agents to trigger status epilepticus (SE) [18]. These models exhibit a high mortality rate, particularly during the SE period, and result in significant neuronal loss across the cortex [19,20]. In contrast, the kindling model employs repeated electrical stimulation to induce seizures. However, it is challenging to evoke spontaneous seizures with this model, often necessitating electrical stimulation to precipitate seizures [21]. Kindling also induces neuronal damage, leading to a loss of up to 50% of hippocampal neurons [22]. Another acute model of focal cortical seizures involves administering repeated doses of 4-aminopyridine (4-AP). This approach induces repetitive seizures that originate at the injection site and spread to the contralateral hemisphere, leading to tonic-clonic seizures that escalate in severity over time. Although the seizures provoked by 4-AP are severe, their effects are transient [23]. Conversely, the TeNT model can be employed to replicate either temporal lobe epilepsy or neocortical epilepsy. This is achieved by administering an intracerebral injection of TeNT to induce seizures without triggering status epilepticus. Additionally, this model has the capability to induce chronic interictal spiking following a single neocortical TeNT injection without any seizure development. Similar to the 4-AP model, contralateral spread to the opposite hemisphere is also seen with TeNT. One notable advantage of the TeNT model lies in its ability to maintain cortical cytoarchitecture. Neurons remain intact following toxin injection, thereby reducing the potential interference of neuron loss and neuroinflammation associated with cell death [24,25,26,27].

Here, we present the development of the TeNT animal model for studying and developing therapeutics for interictal spiking. Additionally, we explore novel therapeutic approaches for interictal spiking in preclinical studies.

## 2. Interictal Spiking Has Highly Reproducible Propagation Patterns and Adversely Affects Cognition and Behavior

Preclinical investigations have revealed that interictal spiking often appears prior to the development of subsequent seizure activity following brain injury [28]. Spike rates can also serve as valuable biomarkers, aiding in seizure prediction. However, it remains unclear whether spikes directly promote or inhibit seizures. Long-term electroencephalography recordings reveal a significant correlation between the probability distributions of spikes and seizures, indicating interconnected processes. A surprising decrease in spike rate prior to seizures suggests spikes may not directly trigger seizures. Nevertheless, the parallel distributions imply potential interactions, with spikes potentially inhibiting seizures or signaling impending seizure activity [29].

Spikes are not isolated but propagate throughout the neocortex and hippocampus with highly reproducible patterns [30]. From human intracranial grid recordings, we found that interictal spikes spread throughout the epileptic neocortex in highly stereotypical patterns, revealing consistent and personalized propagation patterns within each patient. Spikes were observed to traverse various frequency bands, providing novel insights into cortical structure and spike dynamics. Local propagation was predominant, with sporadic long-distance transmission hindered by the central sulcus [30]. Surprisingly, brain regions with the highest spike occurrence did not consistently initiate the spikes but received propagating spikes from multiple brain areas [5]. This was true both in the human neocortex and in the hippocampus, where patients with foramen ovale electrodes had interictal spike networks that were remarkably consistent over time and across various frequency bands within the temporal lobe [31]. These networks often showed ‘reverberations’ between nearby brain regions and were closely associated with seizure onset zones and structural lesions. Additionally, only a small subset of mesial temporal spikes were associated with cortical spikes but lacked a distinct pattern of propagation [31].

Interictal spikes, commonly observed in patients diagnosed with epilepsy, are often linked to cognitive and behavioral comorbidities. Research suggests that approximately 50% of spikes may induce temporary cognitive impairment [32,33]. In adults with epilepsy, spikes have been shown to hinder memory retention and word retrieval, delay reaction times, and increase the risk of accidents in virtual driving simulations. Notably, individuals with temporal lobe epilepsy often exhibit a negative correlation between spikes and executive functioning, particularly verbal fluency [34,35,36,37]. Furthermore, a higher spike frequency has been associated with lower intelligence quotient scores, indicating a potential biomarker for cognitive impairment in adult epilepsy patients [38]. In children, spikes can impair arithmetic skills and reduce attention and processing speeds [39]. Centrotemporal spikes originating near the hippocampus have been found to impede both short- and long-term declarative memory abilities [40]. Additionally, children with left-sided spikes may experience more pronounced impairments in reading performance compared to those with right-sided spikes, suggesting a predictive value of spike location for functional deficits [41]. However, persistent spiking-induced neuronal synchrony can disrupt cortical function in distant regions connected to the spike-onset zone, potentially resulting in widespread deficits despite localized spiking [42].

A number of animal models have corroborated these human findings, showing that interictal spikes are associated with impairments in short-term memory, spatial memory, and object recognition [43,44]. In a rodent model of temporal lobe epilepsy, spikes were specifically found to impair memory retrieval without affecting memory encoding or maintenance [45]. Spikes induced during early life can have lasting effects into adulthood, leading to impaired performance on spatial memory tasks, reduced long-term potentiation, and persistent deficits in sociability and attention [46]. In our studies, we refined a chronic rat model that predominantly induces interictal spiking following the injection of TeNT into the rat somatosensory cortex. Animals exhibiting interictal spiking are hyperactive compared to control animals. Furthermore, the level and type of locomotor activity correlate with the intensity and location of their spiking [47,48]. In a recent investigation, we established that treatment of animals with MAPK inhibitors following TeNT injection into the somatosensory cortex resulted in reduced interictal spikes, alleviated microglial activation, mitigated the loss of inhibitory neurons, and led to improved cognition [49]. Interestingly, a significant decrease in high-amplitude, short-duration spikes in animals treated with MAPK inhibitors demonstrated enhanced spatial memory performance on the Barnes maze.

## 3. Tetanus Toxin: An Ideal Model to Study the Development of Focal Epilepsy and for Drug Development

The TeNT model holds several advantages over other experimental models of chronic focal epilepsy because of its ability to induce spontaneous, chronic seizures and epileptiform activity in numerous higher regions of the brain without causing substantial neuronal loss, tissue damage, or other significant alterations to neurological structures [24,50,51]. Chronic, focal neocortical epilepsy can be produced by direct injection of TeNT into the gray matter of specific brain regions, resulting in spontaneous recurrent seizures and epileptiform activities [52]. Seizures can be induced in a dose-dependent manner with TeNT and are typified by myoclonic movements of the forelimbs [53]. The toxin appears to exert its effects by binding to neuronal cells through an interaction in the plasma membrane. Its light chain functions as a zinc protease that selectively targets vesicle-associated membrane protein (VAMP; synaptobrevin), while the heavy chain facilitates its uptake into neurons. Since VAMP is crucial for synaptic transmission, its proteolysis by TeNT disrupts synaptic function [54,55,56]. The toxin moves by anterograde axonal transport to synapses, where it acts to presynaptically block the release of inhibitory neurotransmitters, most notably gamma-aminobutyric acid (GABA) and glycine [57]. This action reduces inhibitory control over motor and sensory neurons, leading to increased neuronal excitability. The imbalance between excitatory neurotransmitters (e.g., glutamate) and inhibitory neurotransmitters (e.g., GABA) may result in epileptic seizures [58,59,60]. Additionally, tetanus toxin can activate microglia, triggering inflammation that further disrupts inhibitory control and enhances the potential for epileptic activity [49].

While these are acute effects of the toxin, TeNT’s long-lasting effects are what enable it to be useful in studying chronic epilepsy development following a single injection in a specific brain region. The earliest study that used TeNT demonstrated that it was an effective agent capable of producing a chronically active, focal discharging lesion in the cortical brain regions of dogs. Major recurrent convulsions and abnormal spiking first appeared in the tetanus-injected dogs between two and seven days post-injection and persisted, in some cases, for over a month [61]. Subsequent studies repeatedly demonstrated the successful application of TeNT to numerous telencephalic regions, including the cat motor cortex [62] and rat hippocampus [24]. Injection of TeNT into the rat’s hippocampus resulted in alterations in brain function and the occurrence of spontaneous seizures in the absence of status epilepticus. Approximately one month after administration, spike-wave activity, characterized by frequencies ranging from 3 to 20 Hz, was observed in the EEG [52]. Following the injection of a small amount of TeNT into the rat neocortex, excessive synchronization of neuronal activity occurred. This manifested as spontaneous paroxysmal field potentials and/or evoked all-or-none population burst discharges in parietal and temporal areas of both the injected and contralateral hemispheres, starting as early as 16 h after injection and persisting up to 7 months [63]. In another mouse model, unilateral injection of TeNT into the visual cortex was monitored using two electrodes placed into either side of the visual cortex for one-hour local field potential (LFP) recordings. Recordings were performed three days after the surgery and continued for up to 45 days. Utilizing nonlinear time series analysis methods, it was demonstrated that TeNT injection into one hemisphere significantly influenced the local electrical activity of neural populations in both the injected and the opposite hemisphere [64,65].

**Figure 1:**
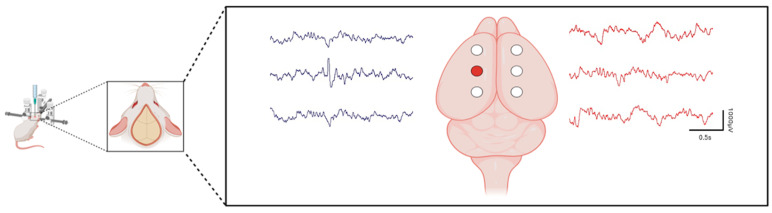
Electrode placement and spike localization in a rat model of interictal spiking generated from a single TeNT injection into the somatosensory cortex. Schematic of the stereotactic surgery illustrates the placement of six recording electrodes. Sample EEG traces demonstrate the localization of interictal spikes. The field is most prominent over the injection site but is also detectable to a lesser extent in adjacent leads. The electrode positioned over the injection site is marked by a red circle. Scale bars: 0.5 s (horizontal) × 1000 µV (vertical).

We have established and characterized a TeNT model of chronic, focal interictal spiking in rats optimized for long-term video EEG recordings and behavioral analyses (see Figure 1). This model involves the injection of TeNT into the somatosensory cortex of rats and results in a prolonged latent period spanning several weeks before the onset of spontaneous spikes and seizures. During this latent period, there is a gradual development and progression of interictal spiking (from 10 spikes per hour on day 5, increasing to 180 spikes per hour after 30 days), providing an opportunity to investigate the development and clinical consequences of neocortical spiking in the absence of seizures. Unlike other animal models of epilepsy that have significant and often diffuse brain injuries, this model offers a unique vantage point to view the specific effects of both spike and subsequent seizure development. This model nicely replicates the latency period observed in human focal epilepsy before the onset of spontaneous seizures [50].

Depending on the cortical injection site, the TeNT model can be used to generate either spikes alone or spikes and seizures [50,66]. Using the exact same protocol for injecting TeNT and epidural electrode implantation, we compared the effects of somatosensory and motor cortex injection sites. Unlike the somatosensory cortex-injected animals, which only displayed spiking activity without the presence of spontaneous seizures, the motor cortex-injected animals developed both spikes and spontaneous seizures. These results confirm the observations from previous studies using rat models involving TeNT injections into the somatosensory cortex and motor cortex [48]. While both somatosensory cortex- and motor cortex-injected animals exhibited abnormal spiking, it occurred in different brain regions. Contralateral spiking was present in both somatosensory cortex and motor cortex-injected animals. However, a primary spiking focus was more likely to develop contralaterally to the injection site in motor cortex-injected animals [48]. Not surprisingly, animals with spiking-only or spiking-plus seizures also displayed marked differences in behavior. While somatosensory cortex-injected animals showed hyperactive behaviors, those with motor cortex injections and seizures were hypoactive. In both models, spikes were not restricted to the injection site, but spiking in specific brain regions correlated with specific locomotor behaviors.

In summary, depending on the location of the injection site and the amount of TeNT injected, the TeNT model offers wide-ranging opportunities to study the development of both interictal spikes and seizures. An important take-home message from these studies is that while the toxin has early effects that appear to be localized to the injection site, the downstream epileptogenic process can involve both this site and other ipsilateral as well as contralateral network sites throughout the brain.

## 4. Identification of Novel Therapeutics against Interictal Spiking from Human Epileptic Tissues

Studies on the human interictal spiking cortex of patients undergoing surgery for refractory seizures offer a unique opportunity to identify novel therapeutic targets for spiking from human tissues removed to control seizures [28]. Differential gene expression from human epileptic, spiking, and non-spiking brain regions has generated a ‘pipeline’ of novel therapeutic targets that selectively target interictal spiking.

Ontological analysis of these differentially expressed genes pointed to the activation of the mitogen-activated protein kinase pathway (MAPK) in the superficial layers of the neocortex (layers 1–3) in the high-spiking human cortex [67], suggesting inhibiting MAPK signaling as a potential therapeutic intervention [28,66,67]. In addition, small patches of an endogenous MAPK inhibitor gene called dual specificity phosphatase 4 (DUSP4) were expressed in the same layers 2/3 of the epileptic neocortex and were associated with a significant reduction in MAPK genes at these sites. Consistently, in vitro studies on the human neuronal-like cell line (Sh-SY5Y) demonstrated that DUSP4 acts as a potent and transient MAPK antagonist, is induced rapidly after repeated depolarizations, and is dependent on MAPK signaling. Overall, these findings suggest that DUSP4 functions as an activity-dependent, negative feedback inhibitor of MAPK signaling expressed in focal brain regions, potentially serving as a localized, endogenous inhibitor for the propagation of epileptic signaling [68]. Bioinformatic and genomic studies have also revealed a group of long non-coding RNAs (lncRNAs) co-regulated with MAPK genes in the human epileptic neocortex. Some of these lncRNAs were directly regulated by MAPK signaling, while other lncRNAs induced in spiking regions specifically downregulated the expression of their antisense coding genes [69]. Building upon these findings, our investigations extended into experimental validation studies using the TeNT model [49].

## 5. Testing Potential Therapeutics That Target Interictal Spiking Using the TeNT Model

The key to the successful translation of potential therapeutics to patients requires animal models that closely parallel the human condition. We have found that the TeNT model of interictal spiking closely parallels human neocortical epilepsy [49,66]. The TeNT model shows a gradual development of spiking with activation of MAPK/CREB signaling pathways in the same neuronal lamina (layers 2/3) as well as many of the downstream genes found in human cortical spiking brain regions. Using this model, a highly specific MAPK inhibitor effectively prevented the onset of epileptic discharges following TeNT injection into the neocortex, with no discernible side effects on brain activity. This experimental outcome underscores the pivotal involvement of the MAPK signaling pathway in the genesis of epileptic activity and highlights the potential of MAPK inhibition as a promising therapeutic intervention [66].

The effects of narcotics on seizure or spiking activity are evident both in the hemisphere where the seizures or spiking are induced and in the contralateral hemisphere. In a more recent study using a different MAPK inhibitor, we demonstrated that a one-week administration of CI-1040, targeting MAP2K, reduced spike frequency and was associated with improved behavior when given either directly after the TeNT administration or two weeks later [49]. In addition to reducing interictal spike occurrence, the treatment prevented the loss of inhibitory neurons and other neuronal and microglial changes seen both in human cortical spiking regions and in the TeNT model. Importantly, animals treated with CI-1040 exhibited a significant decrease in high-amplitude, short-duration spikes, which positively correlated with improved spatial memory performance on the Barnes maze [49].

## 6. Conclusions

The TeNT animal epilepsy model can generate a diverse repertoire of conditions that lead to the development of spike and seizure networks. Many of the changes seen in this animal model closely parallel the human condition and help us enhance our understanding of epilepsy and develop new therapeutics. Interictal spikes have been linked to cognitive and behavioral impairments in patients with epilepsy and those with non-epileptic psychiatric disorders. However, understanding the specific role of interictal spikes has been challenging due to most animal models exhibiting both spikes and seizures, hindering the study of spiking’s relationship with behavior in a seizure-free context. To address this, our laboratory has developed a rat model of interictal spiking wherein rats display high levels of spiking activity without seizures. This model utilizes tetanus toxin injected into the somatosensory cortex to induce consistent and reproducible spikes. Although some anti-seizure drugs have demonstrated the ability to suppress spikes, there is currently a lack of drugs that explicitly target spike activity. Identification of potential therapeutic targets from differential gene expression profiles originating from human epileptic brain-spiking brain regions paired with a highly comparable TeNT model can enable the ability to test promising new therapeutics for clinical translation.

## References

[1] Pillai J., Sperling M.R. (2006). Interictal EEG and the diagnosis of epilepsy. Epilepsia.

[2] Tong J., Ji T., Liu T., Liu J., Chen Y., Li Z., Lu N., Li Q. (2024). Efficacy and safety of six new antiseizure medications for adjunctive treatment of focal epilepsy and epileptic syndrome: A systematic review and network meta-analysis. Epilepsy Behav..

[3] Sundqvist A. (2002). Epilepsy: A clinical diagnostic overview. Eur. J. Pain.

[4] Korzeniewska A., Cervenka M.C., Jouny C.C., Perilla J.R., Harezlak J., Bergey G.K., Franaszczuk P.J., Crone N.E. (2014). Ictal propagation of high frequency activity is recapitulated in interictal recordings: Effective connectivity of epileptogenic networks recorded with intracranial EEG. Neuroimage.

[5] Maharathi B., Wlodarski R., Bagla S., Asano E., Hua J., Patton J., Loeb J.A. (2019). Interictal spike connectivity in human epileptic neocortex. Clin. Neurophysiol..

[6] De Curtis M., Avanzini G. (2001). Interictal spikes in focal epileptogenesis. Prog. Neurobiol..

[7] Salinsky M., Kanter R., Dasheiff R.M. (1987). Effectiveness of multiple EEGs in supporting the diagnosis of epilepsy: An operational curve. Epilepsia.

[8] Kim D.W., Kim H.K., Lee S.K., Chu K., Chung C.K. (2010). Extent of neocortical resection and surgical outcome of epilepsy: Intracranial EEG analysis. Epilepsia.

[9] Inui K., Motomura E., Okushima R., Kaige H., Inoue K., Nomura J. (1998). Electroencephalographic findings in patients with DSM-IV mood disorder, schizophrenia, and other psychotic disorders. Biol. Psychiatry.

[10] Tharp B.R. (2004). Epileptic encephalopathies and their relationship to developmental disorders: Do spikes cause autism?. Ment. Retard. Dev. Disabil. Res. Rev..

[11] Hughes J.R. (1996). A review of the usefulness of the standard EEG in psychiatry. Clin. Electroencephalogr..

[12] Bridgers S.L. (1987). Epileptiform abnormalities discovered on electroencephalographic screening of psychiatric inpatients. Arch. Neurol..

[13] Kim H.L., Donnelly J.H., Tournay A.E., Book T.M., Filipek P. (2006). Absence of seizures despite high prevalence of epileptiform EEG abnormalities in children with autism monitored in a tertiary care center. Epilepsia.

[14] Richer L.P., Shevell M.I., Rosenblatt B.R. (2002). Epileptiform abnormalities in children with attention-deficit-hyperactivity disorder. Pediatr. Neurol..

[15] Volnova A., Tsytsarev V., Ganina O., Vélez-Crespo G.E., Alves J.M., Ignashchenkova A., Inyushin M. (2022). The anti-epileptic effects of carbenoxolone in vitro and in vivo. Int. J. Mol. Sci..

[16] Temkin N.R. (2009). Preventing and treating posttraumatic seizures: The human experience. Epilepsia.

[17] Crunelli V., Lőrincz M.L., McCafferty C., Lambert R.C., Leresche N., Di Giovanni G., David F. (2020). Clinical and experimental insight into pathophysiology, comorbidity and therapy of absence seizures. Brain.

[18] Rusina E., Bernard C., Williamson A. (2021). The kainic acid models of temporal lobe epilepsy. Eneuro.

[19] Lévesque M., Avoli M. (2013). The kainic acid model of temporal lobe epilepsy. Neurosci. Biobehav. Rev..

[20] Covolan L., Mello L. (2000). Temporal profile of neuronal injury following pilocarpine or kainic acid-induced status epilepticus. Epilepsy Res..

[21] Jefferys J.G. (2003). Models and mechanisms of experimental epilepsies. Epilepsia.

[22] Pitkänen A., Sutula T.P. (2002). Is epilepsy a progressive disorder? Prospects for new therapeutic approaches in temporal-lobe epilepsy. Lancet Neurol..

[23] Couturier N.H., Durand D.M. (2020). Comparison of fiber tract low frequency stimulation to focal and ANT stimulation in an acute rat model of focal cortical seizures. Brain Stimul..

[24] Mellanby J., George G., Robinson A., Thompson P. (1977). Epileptiform syndrome in rats produced by injecting tetanus toxin into the hippocampus. J. Neurol. Neurosurg. Psychiatry.

[25] Louis E.D., Williamson P.D., Darcey T.M. (1990). Chronic focal epilepsy induced by microinjection of tetanus toxin into the cat motor cortex. Electroencephalogr. Clin. Neurophysiol..

[26] Jefferys J., Evans B., Hughes S., Williams S. (1992). Neuropathology of the chronic epileptic syndrome induced by intrahippocampal tetanus toxin in rat: Preservation of pyramidal cells and incidence of dark cells. Neuropathol. Appl. Neurobiol..

[27] Brace H., Jefferys J., Mellanby J. (1985). Long-term changes in hippocampal physiology and learning ability of rats after intrahippocampal tetanus toxin. J. Physiol..

[28] Kirchner A., Dachet F., Loeb J.A. (2020). Identifying targets for preventing epilepsy using systems biology of the human brain. Neuropharmacology.

[29] Dachet F., Bagla S., Keren-Aviram G., Morton A., Balan K., Saadat L., Valyi-Nagy T., Kupsky W., Song F., Dratz E. (2015). Predicting novel histopathological microlesions in human epileptic brain through transcriptional clustering. Brain.

[30] Maharathi B., Loeb J.A., Patton J. Central sulcus is a barrier to causal propagation in epileptic networks. Proceedings of the 2019 41st Annual International Conference of the IEEE Engineering in Medicine and Biology Society (EMBC).

[31] Maharathi B., Patton J., Serafini A., Slavin K., Loeb J.A. (2021). Highly consistent temporal lobe interictal spike networks revealed from foramen ovale electrodes. Clin. Neurophysiol..

[32] Nicolai J., Trenité D.K.-N. (2011). Interictal discharges and cognition. Epilepsy Behav..

[33] Holmes G.L., Lenck-Santini P.-P. (2006). Role of interictal epileptiform abnormalities in cognitive impairment. Epilepsy Behav..

[34] Ung H., Cazares C., Nanivadekar A., Kini L., Wagenaar J., Becker D., Krieger A., Lucas T., Litt B., Davis K.A. (2017). Interictal epileptiform activity outside the seizure onset zone impacts cognition. Brain.

[35] Nirkko A.C., Bernasconi C., von Allmen A., Liechti C., Mathis J., Krestel H. (2016). Virtual car accidents of epilepsy patients, interictal epileptic activity, and medication. Epilepsia.

[36] Kleen J.K., Scott R.C., Holmes G.L., Roberts D.W., Rundle M.M., Testorf M., Lenck-Santini P.-P., Jobst B.C. (2013). Hippocampal interictal epileptiform activity disrupts cognition in humans. Neurology.

[37] Dinkelacker V., Xin X., Baulac M., Samson S., Dupont S. (2016). Interictal epileptic discharge correlates with global and frontal cognitive dysfunction in temporal lobe epilepsy. Epilepsy Behav..

[38] Meekes J., Jennekens-Schinkel A. (2018). Effects of interictal epileptiform discharges on cognition. J. Pediatr. Epilepsy.

[39] Cheng D., Yan X., Xu K., Zhou X., Chen Q. (2020). The effect of interictal epileptiform discharges on cognitive and academic performance in children with idiopathic epilepsy. BMC Neurol..

[40] Verrotti A., Filippini M., Matricardi S., Agostinelli M.F., Gobbi G. (2014). Memory impairment and Benign Epilepsy with centrotemporal spike (BECTS): A growing suspicion. Brain Cogn..

[41] Trenité D.K.N., Siebelink B., Berends S., Van Strien J., Meinardi H. (1990). Lateralized effects of subclinical epileptiform EEG discharges on scholastic performance in children. Epilepsia.

[42] Landi S., Petrucco L., Sicca F., Ratto G.M. (2019). Transient cognitive impairment in epilepsy. Front. Mol. Neurosci..

[43] Shatskikh T.N., Raghavendra M., Zhao Q., Cui Z., Holmes G.L. (2006). Electrical induction of spikes in the hippocampus impairs recognition capacity and spatial memory in rats. Epilepsy Behav..

[44] Stafstrom C.E. (2010). Interictal spikes: Memories forsaken. Epilepsy Curr..

[45] Kleen J.K., Scott R.C., Holmes G.L., Lenck-Santini P.P. (2010). Hippocampal interictal spikes disrupt cognition in rats. Ann. Neurol. Off. J. Am. Neurol. Assoc. Child Neurol. Soc..

[46] Khan O.I., Zhao Q., Miller F., Holmes G.L. (2010). Interictal spikes in developing rats cause long-standing cognitive deficits. Neurobiol. Dis..

[47] Serafini R., Dettloff S., Loeb J. (2016). Neocortical slices from adult chronic epileptic rats exhibit discharges of higher voltages and broader spread. Neuroscience.

[48] Geraghty J.R., Senador D., Maharathi B., Butler M.P., Sudhakar D., Smith R.A., Wu Y., Loeb J.A. (2021). Modulation of locomotor behaviors by location-specific epileptic spiking and seizures. Epilepsy Behav..

[49] Smith R.A., Mir F., Butler M.P., Maharathi B., Loeb J.A. (2024). Spike-induced cytoarchitectonic changes in epileptic human cortex are reduced via MAP2K inhibition. Brain Commun..

[50] Barkmeier D., Loeb J. (2009). An animal model to study the clinical significance of interictal spiking. Clin. EEG Neurosci..

[51] Crisp D.N., Cheung W., Gliske S.V., Lai A., Freestone D.R., Grayden D.B., Cook M.J., Stacey W.C. (2020). Quantifying epileptogenesis in rats with spontaneous and responsive brain state dynamics. Brain Commun..

[52] Finnerty G., Jefferys J. (2002). Investigation of the neuronal aggregate generating seizures in the rat tetanus toxin model of epilepsy. J. Neurophysiol..

[53] Mellanby J., Hawkins C., Mellanby H., Rawlins J., Impey M. (1984). Tetanus toxin as a tool for studying epilepsy. J. Physiol..

[54] van Heyningen S. (1980). Tetanus toxin. Pharmacol. Ther..

[55] Louis E.D., Williamson P.D., Darcey T. (1987). Experimental models of chronic focal epilepsy: A critical review of four models. Yale J. Biol. Med..

[56] Ferecskó A.S., Jiruska P., Foss L., Powell A.D., Chang W.-C., Sik A., Jefferys J.G. (2015). Structural and functional substrates of tetanus toxin in an animal model of temporal lobe epilepsy. Brain Struct. Funct..

[57] Calvo A.C., Oliván S., Manzano R., Zaragoza P., Aguilera J., Osta R. (2012). Fragment C of tetanus toxin: New insights into its neuronal signaling pathway. Int. J. Mol. Sci..

[58] Benke T.A., Swann J. (2004). The tetanus toxin model of chronic epilepsy. Recent Adv. Epilepsy Res..

[59] Curtis D., Felix D., Game C., McCulloch R. (1973). Tetanus toxin and the synaptic release of GABA. Brain Res..

[60] Empson R., Amitai Y., Jefferys J., Gutnick M. (1993). Injection of tetanus toxin into the neocortex elicits persistent epileptiform activity but only transient impairment of GABA release. Neuroscience.

[61] Carrea R., Lanari A. (1962). Chronic effect of tetanus toxin applied locally to the cerebral cortex of the dog. Science.

[62] Brooks V., Asanuma H. (1962). Action of tetanus toxin in the cerebral cortex. Science.

[63] Brener K., Amitai Y., Jefferys J.G., Gutnick M.J. (1991). Chronic epileptic foci in neocortex: In vivo and in vitro effects of tetanus toxin. Eur. J. Neurosci..

[64] Vannini E., Caleo M., Chillemi S., Di Garbo A. (2017). Dynamical properties of LFPs from mice with unilateral injection of TeNT. Biosystems.

[65] Mainardi M., Pietrasanta M., Vannini E., Rossetto O., Caleo M. (2012). Tetanus neurotoxin–induced epilepsy in mouse visual cortex. Epilepsia.

[66] Barkmeier D.T., Senador D., Leclercq K., Pai D., Hua J., Boutros N.N., Kaminski R.M., Loeb J.A. (2012). Electrical, molecular and behavioral effects of interictal spiking in the rat. Neurobiol. Dis..

[67] Beaumont T.L., Yao B., Shah A., Kapatos G., Loeb J.A. (2012). Layer-specific CREB target gene induction in human neocortical epilepsy. J. Neurosci..

[68] Kirchner A., Bagla S., Dachet F., Loeb J. (2020). DUSP4 appears to be a highly localized endogenous inhibitor of epileptic signaling in human neocortex. Neurobiol. Dis..

[69] Kirchner A., Dachet F., Lipovich L., Loeb J.A. (2023). Activity-dependent non-coding RNA MAPK interactome of the human epileptic brain. Non Coding RNA.

